# Successful *en bloc* transplantation of pediatric deceased donor kidneys with grade 1 injury

**DOI:** 10.4103/0971-4065.59341

**Published:** 2009-10

**Authors:** P. Modi, S. J. Rizvi, H. L. Trivedi

**Affiliations:** Department of Urology and Transplantation Surgery, Dr. H. L. Trivedi Institute of Transplantation Sciences, India; 1 Department of Smt. G R Doshi and K M Mehta Institute of Kidney Diseases and Research Centre, Dr. H. L. Trivedi Institute of Transplantation Sciences, India

**Keywords:** Cadaver, deceased donor, *en bloc*, kidney transplantation, pediatric

## Abstract

Kidney transplantation from deceased donors is in its infancy in India. Marginal donors are now accepted by many centers for kidney transplantation. We report a case of procurement of *en bloc* kidneys from a pediatric deceased donor having grade 1 renal injury and transplanted to an adult recipient with a follow up of two years and five months.

## Introduction

Definition of brain death and use of organs for purpose of transplantation was accepted in India in 1994. The Government of Gujarat had accepted the law in 1997 and we had performed the first deceased donor transplantation in the same year. In 2006, we had established intercity cadaver transplant program and procured organs from remote cities.

Organ procurement for purpose of transplantation from deceased pediatric donors is rare in India. We report a case of procurement of both kidneys from a four and half year old child and transplantation *en bloc* to an adult recipient. Procurement of organs was performed in a city 270 km away from our center.

## Case Report

**Figure 1:**
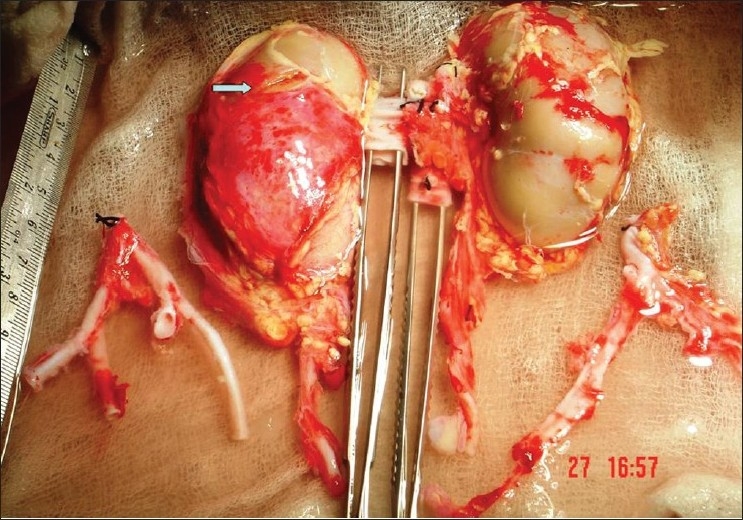
*En bloc* procurement of kidneys with aorta and vena cava. Note the laceration on ventral surface of right kidney

**Figure 2:**
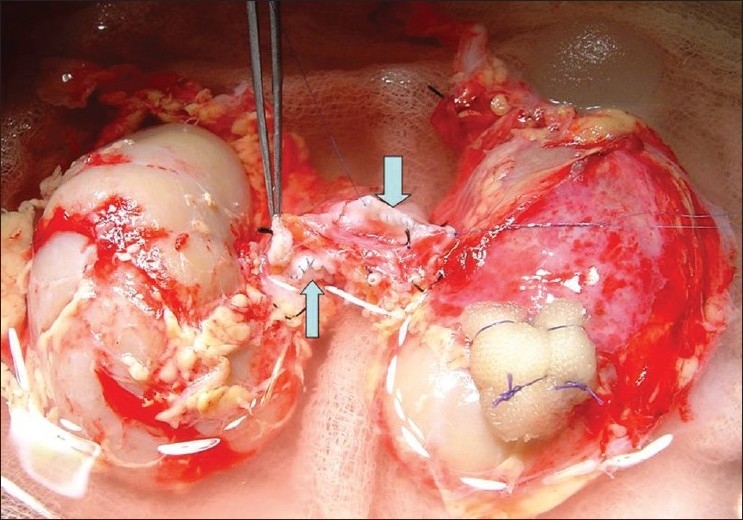
Closed upper ends of aorta (thin arrow) and vena cava (thick arrow). Surgicel® bolster was used for repair of laceration at right kidney upper pole

**Figure 3:**
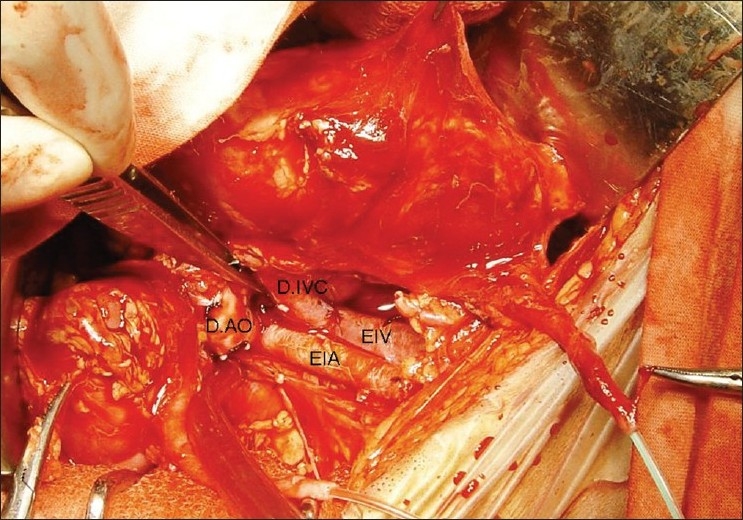
*En bloc* transplantation of both kidneys. D.AO = donor aorta, D.IVC = donor inferior vena cava, EIA = external iliac artery, EIV = external iliac vein

A 5-year-old boy had met with road traffic accident and was declared brain dead. Parents and other nearby relatives gave consent for use of organs for purpose of transplantation. The size of both kidneys on ultrasound examination was 7.8 × 2.5 cm. A 1.2 cm long grade 1 injury was found on the anterior surface of the right kidney. Kidneys were perfused *in situ* after procurement by clamping the suprarenal aorta using Ringer's lactate solution [Figure 1] and packed *en bloc* for transport and transplantation. On bench, the laceration was repaired by Surgicel® bolster and upper end of aorta and vena cava were sutured by 4/0 and 5/0 polypropylene sutures respectively [Figure 2]. The lower end of donor aorta and vena cava were anastomosed to recipient's external iliac vessels and both kidneys were placed extraperitoneally in a 42-year-old female recipient who had renal failure due to chronic glomerulonephritis [Figure 3]. Both ureters were implanted by modified Lich-Gregoir method over 4.5 Fr size double ‘J’ stents. Drain tube was placed and wound was closed in layers.

## Results

The cold ischemic time was 11 h and 30 min and re-warming time was 35 min. Drain tube was removed after 48 h of surgery. Urine output was established promptly and patient had serum creatinine value of 0.90 mg/dl at the time of discharge on fifteenth post operative day. Antithymocyte globulin, Rituximab and methyl prednisolone were given as induction agents and she was maintained on cyclosporine, mycophenolate mofetil and prednisolone. During follow-up after 2 years and 5 months post-transplant, her serum creatinine value is 0.99 mg/dl with normal blood flow pattern on doppler ultrasound examination and each kidney size is 11 × 4.5 cm. She does not have proteinuria or hypertension. The estimated GFR was 88 ml/min/1.73 m^2^.

### Comments

Single kidney versus *en bloc* transplantation of kidneys from young deceased donor is controversial. Single kidney transplantation is more likely associated with technical complications and long term graft loss due to hyperfiltration injury to the graft. To overcome such problems, *en bloc* transplantation is carried out.[1] More recent report, however, has shown that regular use of Carell's patch or aortic conduit has significantly reduced the vascular complications with single pediatric kidney transplantation though delayed graft function and reduction in glomerular filtration rate at the end of one year is significantly higher in patients receiving single kidney than *en bloc* kidneys from a pediatric donor.[2] Injury to the kidney could be present at the time of accident or later on during procurement of the organ. Kidney parenchymal laceration is described as grade 1 injury. Such injury should be repaired and organ should not be discarded.

Kidneys from small donors may be an increased risk for late graft failure if they are transplanted into large recipients, and therefore, the relative size of the donor and recipient should be taken into account. Allocation of single kidney to pediatric recipient is logical and gives good long term result.[3] However, an experienced pediatric transplant surgeon is required to avoid technical problems during vascular anastomosis and subsequent graft loss.

Clinical and animal studies have demonstrated that transplanted kidneys will increase in size and function over time, provided acute rejection does not ensue.[4] Kidneys are hypertrophied and minimal or no hyperplasia could occur.

This recipient had positive lymphocyte cross-match activity found during previous several occasions; only at this time her lymphocyte cross-match was negative. However, to protect allograft from both donor nonspecific and donor specific antibodies, we used rituximab as induction agent. Cyclosporine- or tacrolimus-based regime in recipient of pediatric kidneys fare better than historical group of azathioprime and prednisolone based regime. Nephrotoxicity of both these drugs are an issue for pediatric kidneys. Cyclosporine blunts adaptive compensatory hypertrophy; however, it occurs in lesser degree in younger kidneys.[5]

## Conclusion

Grade 1 injured kidney from deceased donor should be used for transplantation. *en bloc* kidney transplantation from pediatric deceased donor with cyclosporine based immuno-suppression regime gives a good intermediate term graft survival. To our knowledge, this is the first case report from India to use pediatric kidneys *en bloc* for transplantation.
